# Supplementary material for: Role of framework mutations and antibody flexibility in the evolution of broadly neutralizing antibodies
Source: eLife. 2018 Feb 14;7:e33038. doi: 10.7554/eLife.33038 (PMC5828663; doi:10.7554/eLife.33038)
Supplement: Supplementary file 1. — Values are given if they are constant in all simulations presented here. Values of parameters that were changed for different simulations are given in Supplementary file 2. [file elife-33038-supp1.docx]

|  | Description | Parameter | Value |
| --- | --- | --- | --- |
| Somatic hypermutation | Probability of a mutation in either CDR or FWR per round of division | p_mut | 0.20 |
|  | Probability that a mutation lies in the CDR | p_CDR | 0.85 |
|  | Probability that a CDR mutation is lethal | p_CDR_lethal | 0.30 |
|  | Probability that a CDR mutation is silent | p_CDR_silent | 0.50 |
|  | Probability that a CDR mutation affects affinity | p_CDR_affect | 0.20 (= 1 – p_CDR_lethal – p_CDR_silent) |
|  | Probability that an affinity-affecting CDR mutation affects the variable region | p_var (called λ in main text) | varies |
|  | Probability that an affinity-affecting CDR mutation affects the conserved region | p_cons | varies (= 1 – p_var) |
|  | Mean of shifted lognormal distribution for effect of CDR mutation on binding energy | mu | 1.9 |
|  | STD of shifted lognormal distribution for effect of CDR mutation on binding energy | sigma | 0.5 |
|  | Shift of shifted lognormal distribution for effect of CDR mutation on binding energy | o | 3.0 |
|  | Probability that a mutation lies in the FWR | (unnamed in code, = 1 – p_CDR) | 0.15 (= 1 – p_CDR) |
|  | Probability that a FWR mutation is lethal | p_FR_lethal | 0.80 |
|  | Probability that a FWR mutation is silent | p_FR_silent | 0 |
|  | Probability that a FWR mutation affects flexibility | p_FR_affect | 0.20 (= 1 – p_FR_lethal – p_FR_silent) |
|  | Mean of normal distribution for change in *Q* due to flexibility-affecting mutation | (unnamed in code) | 0 |
|  | STD of normal distribution for change in *Q* due to flexibility-affecting mutation | sigmaQ | 0.08 |
| Binding | Starting value of *E_c_* for new B cells | Ec_start | varies |
|  | Pseudo inverse temperature | energy_scale | 0.07 |
|  | Generic binding energy *E_0_* | E0 | varies |
|  | Antigen concentration | conc | varies |
| GC dynamics | Probability that a B cell is recycled after selection | p_recycle | 0.70 |
|  | Probability that a B cell exits the germinal center after selection | p_exit | 0.30 (= 1 – p_recycle) |
|  | Fraction of B cells that successfully bound antigen that receive T cell help | help_cutoff | 0.70 |
|  | Number of founder B cells | nb_founders | 3 |
|  | Maximum number of B cells in GC | GC_size_max | 1536 |
|  | Maximum number of GC cycles | nb_cycle_max | 250 |
|  |  |  |  |
