# Supplementary material for: Role of framework mutations and antibody flexibility in the evolution of broadly neutralizing antibodies
Source: eLife. 2018 Feb 14;7:e33038. doi: 10.7554/eLife.33038 (PMC5828663; doi:10.7554/eLife.33038)
Supplement: Supplementary file 2. — Note that the energy values in the simulation code have the opposite sign of the physical energy values, as described in the main text. This is simply a sign convention in the code, and has no bearing on the dynamics of the simulation. [file elife-33038-supp2.docx]

|  | Variable parameters | | | | | |
| --- | --- | --- | --- | --- | --- | --- |
| Simulation condition | nb_Ag | p_var | p_cons | Ec_start | E0 | conc |
| Single antigen | 1 | 0.90 | 0.10 | 0 | 4 | 1.11 |
| Strong E_c_ | 10 | 0.90 | 0.10 | 4 | 4 | 0.98 |
| Weak E_c_ | 10 | 0.90 | 0.10 | 0 | 4 | 1.18 |
| Strong E_c_, low λ | 5 | 0.80 | 0.20 | 4 | 4 | 1.08 |
| Weak E_c_, low λ | 5 | 0.80 | 0.20 | 0 | 4 | 1.21 |
| Strong E_0_, weak E_c_ | 10 | 0.90 | 0.10 | 0 | 6 | 1.11 |
| Weak E_0_, weak E_c_ | 10 | 0.90 | 0.10 | 0 | 2 | 1.23 |
